# Progress test as an assessment for learning approach in an Infectious Diseases Residency Program: a case study

**DOI:** 10.1016/j.bjid.2024.103849

**Published:** 2024-07-17

**Authors:** Bianca Eliza Hoekstra, Cinara Silva Feliciano, Renata Teodoro Nascimento, Valdes Roberto Bollela

**Affiliations:** Universidade de São Paulo – Faculdade de Medicina de Ribeirão Preto, Ribeirão Preto, SP, Brazil

**Keywords:** Progress test, Infectious diseases, Residency training, Formative assessment

## Abstract

Assessment is an essential component for all educational programs and must check competence acquirement while foster and promote learning. Progress Test (PT) is well recognized to assess cognitive knowledge, clinical reasoning and decision making in the clinical context, offering important information about the individual performance and program quality. It is widely used in Brazilian and international medical schools; however, it still has little role in assessing medical residents in Brazil. We present the experience of a PT pilot implementation in an Infectious Diseases residency program over two years. The first, second and third-year residents did four serial exams with 40 multiple choice questions (item)/each. Preceptors were trained on best practices on item writing. All the items were reviewed by a panel of experts and, after approval, included in the item bank. All participants answered a survey on their perceptions about the experience. The final score was higher for the third-year residents in all exam applications. The level of satisfaction was high among the participants, who mentioned the learning opportunity with the exam and the feedback. PT can improve residents’ assessment along the training period and residents’ performance should guide review and improvement of the programs.

Assessment is an essential component of any educational program, defined as a systematic data collection about student learning, using appropriate methods and criteria that can be applied for different purposes: summative, formative and/or informative/diagnostic.^1^, ^2^, ^3^

Different assessment tools have been used in medical education to address the different domains of competency required for a future healthcare professional: cognitive, psychomotor and attitudinal-affective.^2^^,^^3^

The main and most widespread strategy for assessing cognitive skills are multiple choice questions, also called items. When properly elaborated, item exams are valid, reliable and easy to mark.^1^ It is strongly recommended to address more than the memorization of concepts, but the ability to analyze, reasoning and decide based on real and relevant clinical problems.^4^^,^^5^

Among the strategies to assess the cognitive domain, the Progress Test (PT) offers some characteristics that highlight its role in medical education. Usually, it is administered to all students/residents in the medical program at the same time on a regular basis (once or twice a year) throughout the entire academic program.^6^^,^^7^ The exam must sample the relevant knowledge expected for the future medical practice and the ability to use it. The scores provide insights about individual students/residents performances as well as the strengths and weaknesses of the educational program.^8^ This information can be consistently used for individual learn and improvement, at the same time that may guide program evaluation, review and improvement.^6^^,^^7^

Since its implementation in 1970s, it has been increasingly used in medical programs worldwide and new approaches have been created, such as inter-university PT collaborations.^7^ This consortia approach provides means of improving the cost-effectiveness of assessments by sharing larger item banks, item writers, reviewers, and administrators.^9^

Both, an individual school and a consortium PT, should follow the main steps to accomplish its educational role: the definition of a coordination team, the blueprint creation, item writing workshops, item bank construction, panel review creation, timely feedback to participants based on the result analysis, including quality control procedures.^7^

Medical residency programs, based on supervised training in real settings, are the gold standard for medical specialization.^10^ However, there is a central role of technical-scientific knowledge for training and qualifying medical activity.^11^^,^^12^ In this sense, the evaluation and monitoring of the knowledge acquisition during the specialty training is essential. Similarly to the undergraduation use, PT shows a great potential as a formative tool to assess medical residents knowledge acquisition longitudinally, with high rates of feasibility, acceptability and catalytic effect.^13^ The first use of PT in residency training dates from 1999, in the Netherlands.^13^ In Brazil, even though it is widely used in medical undergraduate courses, it is underused in medical residency programs, since it has been implemented only in the Obstetrics and Gynecology and Orthopedics specialization programs.^14^^,^^15^

The Infectious Diseases Residency Programs (ID-RP) do not follow a single assessment pattern in Brazil, and many programs do not have knowledge assessment on a regular basis. Based on the potential benefits of PT as a tool to assess and promote learning among medical residents, it was introduced in ID-RP at Hospital das Clínicas da Faculdade de Medicina de Ribeirão Preto in 2021 as a formative assessment.

The first step of this intervention was to engage stakeholders, that were medical residents’ preceptors, and train them on good quality item writing. The items must have a single best answer, always with a clinical vignette (real and prevalent problem), a clear lead in, a key answer and three homogeneous distractors.^16^ Afterwards, a blueprint (exam map) was created based on the national document that establish the competence matrix for the infectious diseases specialialization.^17^ The covered topics included epidemiology, mechanism of disease, clinical reasoning & hypothesis elaboration (diagnosis), decision making on complementary investigation, management (treatment), health promotion, and disease prevention. A template to guide item writing was developed. Finally, the items were submitted to a panel review and, after the final approval, they were sent to an item bank created on Moodle®.

Medical residents attending the three years of the program, that admits five residents per year, were invited to participate in two tests per year (first and second semester), with 40 items each. The examinations were administered through the University of São Paulo's Moodle® platform, adhering to a predetermined schedule and fixed duration. Test items were distributed randomly to each participant. Prior to the examinations, all residents were duly notified of the formative nature of these assessments, with no provision for pass or fail grading. At the end of the exam, residents received a detailed feedback with all the items commented.

We also asked them to fill a survey about their perceptions on the experience with six structured questions (5-point Likert scale) and three open-ended questions:

Structured questions:

1) Serial assessment with multiple choice questions is a useful tool to test my own knowledge.
2) Results analysis can be used to rectify directions during the training of the specialist before the end of the residency program.
3) The serial assessments reinforced my previous knowledge.
4) I acquired new knowledge through assessments.
5) I consider timely feedback a necessary factor for positive results from serial assessments.
6) The assessments helped me to improve my confidence to carrying out board certification or public tests.

Open-ended questions:

1) What did you like most about this experience?
2) What could be improved in the future?
3) Do you have any improvement suggestions for us?

The results analysis included psychometric analysis of the items, measured by discrimination and difficulty index,^18^ to ensure a better and balanced selection of items for future tests.

The proposal was approved by the hospital ethics committee (number 54,851,221.0.0000.5440).

From 2021 to 2023, 300 reviewed items were added to the bank and first, second and third-year medical residents did four tests. The first-year medical residents did not participate in 2 out of 4 tests (second and third PT) due to practical activities previously scheduled (emergency duty). Therefore, the number of participants was 15 in the first PT, 10 in the second and third PT and 15 in the last exam.

The serial performance of all participants was shown in Fig. 1.

**Fig. 1:**
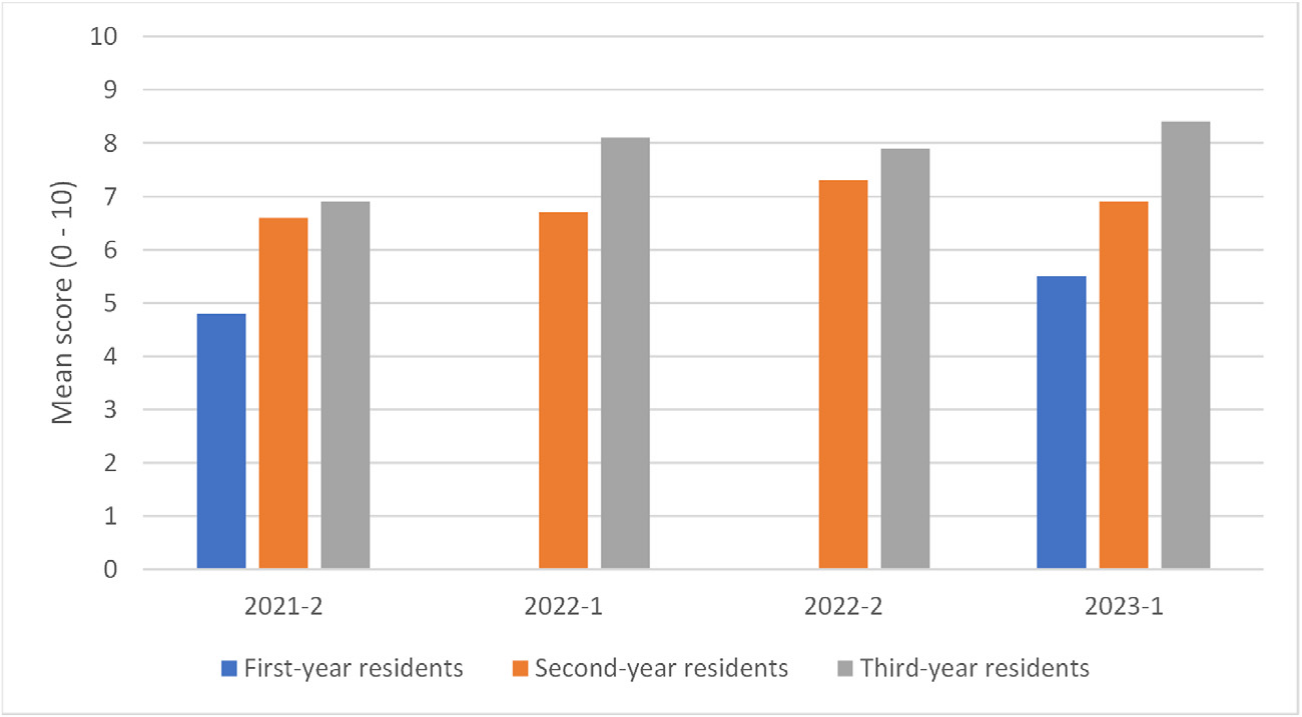
Performance of participants from first, second and third year of ID-RP. 2021-2: second semester of 2021; 2022-1: first semester of 2022; 2022-2: second semester of 2022; 2023-1: first semester of 2023.

One group of residents (five residents) did the four tests as first-year residents (R1) in 2021, second-year in 2022 (R2), and third-year in 2023 (R3). This analysis demonstrates the knowledge improvement trend along the residency program (Fig. 2). The improvement was homogeneous among the addressed topics and tests offered sequential opportunities of learning supported by formative assessment.

**Fig. 2:**
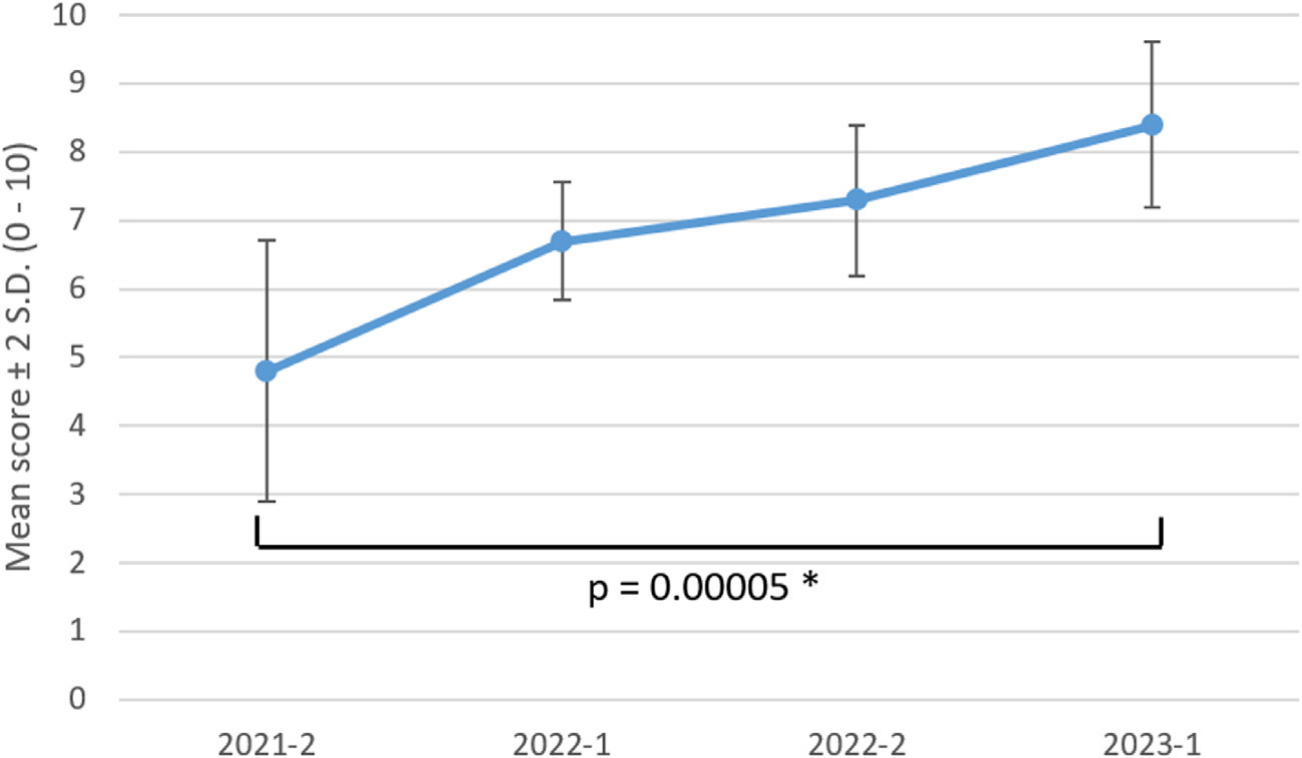
Performance trend of a group of residents (2021 to 2023).2021–2: second semester of 2021; 2022–1: first semester of 2022; 2022–2: second semester of 2022; 2023–1: first semester of 2023. * The difference in the means between the first and last test was statistically significant based on the Student's *t*-test for dependent means.

Regarding the perception survey, most responses were positive (4 or 5 point on Likert scale). Suggestions provided in the open-ended questions included adding face-to-face feedback and increasing the number of annual tests. Most residents reported insecurity in answering questions involving pathologic concepts/findings, which can be used as an opportunity to improve our program.

The relevance of progress test in identifying medical residents’ strengths and weaknesses and providing them with a good basis for making self-assessments and judging learning needs was clear to participants and preceptors. Based on their responses, they felt motivated to remediate areas of weakness.

Our program admits five residents per year, which impairs strong inferences of our results so far, including the results of psychometric analysis of the items. However, this very positive experience should be shared with other programs to stablish, in the future, test consortia. As mentioned, a progress test consortium enables the enhancing of the number of items and reviewers and, consequently, the validity of the test in providing diagnosis not only about individual performance but also about the whole program. A positive example comes from FEBRASGO (Brazilian Federation of Gynecology and Obstetrics), that is currently using unified test results even to classify and qualify its medical residency programs in the country.^19^

Progress test is useful both as assessment and educational intervention, resulting in positive impact on learning outcomes. Thus, it can be a valuable tool to promote constant improvements in ID-RP, contributing to qualify future infectologists to work for the society.

## Conflicts of interest

The authors declare no conflicts of interest.
